# The clinical anatomy of the atrioventricular conduction axis

**DOI:** 10.1093/europace/euae048

**Published:** 2024-02-14

**Authors:** Damián Sánchez-Quintana, Jose-Angel Cabrera, Robert H Anderson

**Affiliations:** Departamento de Anatomía Humana y Biología Celular, Facultad de Medicina, Universidad de Extremadura, Elvas Avenue, Badajoz 06006, Spain; Departamento de Cardiología, Unidad de Arritmias, Hospital Universitario Quirón-Salud Madrid and complejo Hospitalario Ruber Juan Bravo, Universidad Europea de Madrid, Madrid, Spain; Biosciences Institute, Newcastle University, Newcastle upon Tyne, UK

**Keywords:** Atrioventricular conduction axis, Left bundle branch, His bundle pacing, Aortic root

## Abstract

It is axiomatic that the chances of achieving accurate capture of the conduction axis and its fascicles will be optimized by equally accurate knowledge of the relationship of the components to the recognizable cardiac landmarks, and we find it surprising that acknowledged experts should continue to use drawings that fall short in terms of anatomical accuracy. The accuracy achieved by Sunao Tawara (1906) in showing the location of the atrioventricular conduction axis is little short of astounding. Our purpose in bringing this to current attention is to question the need of the experts to have produced such inaccurate representations, since the findings of Tawara have been extensively endorsed in very recent years. The recent studies do no more than point to the amazing accuracy of the initial account of Tawara. At the same time, we draw attention to the findings described in the middle of the 20th century by Ivan Mahaim (1947). These observations have tended to be ignored in recent accounts. They are, perhaps, of equal significance to those seeking specifically to pace the left fascicles of the branching atrioventricular bundle.

## Graphical Abstract

**Graphical Abstract:**
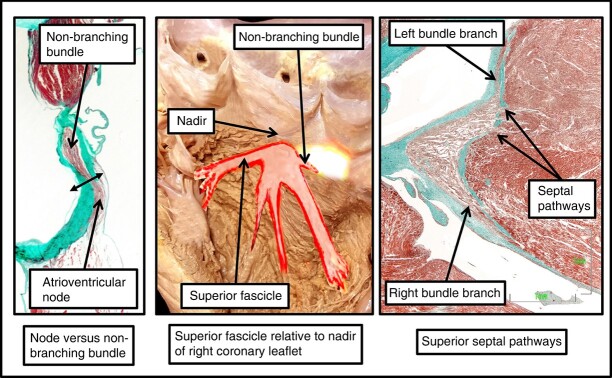
The illustrations show, in the left hand panel, how the atrioventricular conduction axis can be separated into its atrial and ventricular parts at the point of insulation by the fibrous tissues of the atrioventricular junction, indicated by the double-headed black arrow. The middle panel shows our understanding, based on our previous investigations, of the location of the left bundle relative to the aortic root, while the right panel shows an example of the ‘paraspecific’ conduction system described by Ivan Mahaim.

**What’s new?**

- The superior fascicle of the left bundle branch, as had been shown by Sunao Tawara, is closely related to the nadir of the right coronary leaflet of the aortic valve.
- The ‘paraspecific’ system for conduction described by Ivan Mahaim nearly a century ago is not yet fully recognized. It could well provide an alternative means of activating the ventricular myocardium in the setting of a diseased conduction system.

## Introduction

George Santayana, writing in his ‘Life of Reason’ of 1905, commented that ‘those who cannot remember the past are condemned to repeat it.’ The statement is not entirely true in the context of the anatomy of the atrioventricular conduction axis, since it is unlikely that those who seem unaware of the past with regard to this system are likely to repeat the stellar investigation performed by Tawara.^1^ Prior to the studies of Tawara, there had been significant controversy regarding the substrate for normal atrioventricular conduction.^2^ Although His had provided an excellent account of the bundle now described in his name in 1893, in the same year, Stanley Kent, a physiologist working at St Thomas’s Hospital in London, claimed to have demonstrated multiple connections across the parietal atrioventricular junctions. Surprisingly, despite the unequivocal demonstration of the solitary nature of the pathway for conduction by Tawara, Kent still maintained his own beliefs, albeit writing anonymously, well into the early decades of the 20th century.^2^ All of this early research remains topical. This is because current investigators involved in pacing the ventricular components of the conduction axis, particularly its left fascicles, have produced drawings purporting to show the disposition of the bundle and its branches.^3,4^ The significance of the aphorism cited above becomes apparent when even the most cursory examination shows that these drawings have little resemblance to the anatomical relationships as shown by Tawara.^1^ It is axiomatic that the chances of achieving accurate capture of the conduction axis, and equally its fascicles, will be optimized by accurate knowledge of the relationship of the structure to the recognizable cardiac landmarks. We encourage, therefore, the use of anatomically accurate depictions of the location of the conduction system in the pacing literature, since the findings of Tawara have now been extensively endorsed.^5^ We see no excuse for failing to take account of the recent information, which does no more than point to the amazing accuracy of the initial account. At the same time, in the context of pacing the His bundle and its fascicles, we draw attention to the findings described in the middle of the 20th century by Mahaim.^6^ Although largely ignored in most recent accounts, they are particularly significant to those seeking specifically to pace the left fascicles of the branching atrioventricular bundle.

## The findings of Tawara

**Figure 1:**
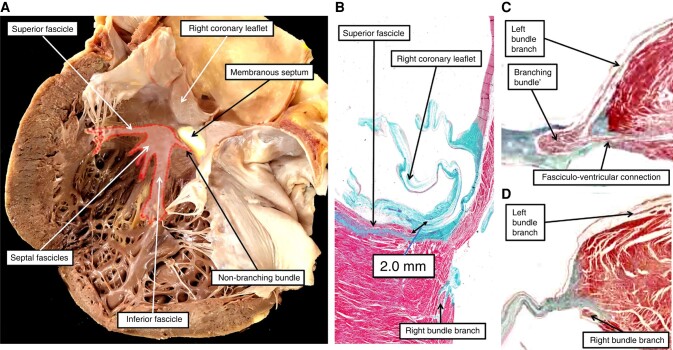
In panel *A*, we have superimposed the location of the fascicles of the left bundle branch on the opened septal surface of the left ventricle. When viewed in attitudinal fashion, the fascicles are located superiorly, septally, and inferiorly. Significantly, the superior fascicle extends to become adjacent to the nadir of the leaflet supported by the right coronary aortic sinus before tracking towards the supero-lateral papillary muscle of the mitral valve. Panel *B* shows how, on occasion, the fascicle can come within 2 mm of the nadir of the leaflet. Panel *C* shows the superior septal connections described initially by Ivan Mahaim. As shown in panel *D*, they make contact with the septal myocardium prior to the take-off of the right bundle branch, with panels *C* and *D* being taken from the same serially sectioned histological dataset.

Tasked by Ludwig Aschoff with finding the substrate for heart failure, Tawara^1^ instead, by careful study of serially sectioned hearts from various species, clarified the arrangement of the atrioventricular conduction axis. He confirmed the finding of His that there was but a solitary myocardial connection across the atrioventricular junctions in the normal heart. More importantly, Tawara showed that the axis commenced in a histologically discrete structure located in the antero-inferior buttress of the atrial septum, which he christened the ‘knoten’. He then showed that the axis, having been insulated from the atrial myocardium by the fibrous tissues of the junction, branched on the crest of the muscular septum, ramifying eventually within the ventricular apical components via the insulated bundle branches. He showed that the point of insulation served to provide a criterion to distinguish between the node and the ventricular components of the axis, with the ventricular axis itself then having non-branching and branching parts. Significantly, he illustrated the fascicles of the left bundle as extending in trifascicular fashion along the septal surface of the left ventricle towards the papillary muscles supporting the mitral valve (*Figure 1A*). His illustration showed clearly the close relationship between the superior fascicle and the nadir of the right coronary leaflet of the aortic valve (*Figure 1B*).

## The findings of Mahaim

Ivan Mahaim was a Belgian national, but practised in Lausanne, Switzerland. He conducted extensive investigations of the ventricular components of the conduction axis, describing his findings in a monograph published in French. He summarized his findings in a short account in which he suggested the presence of a ‘paraspecific’ system for atrioventricular conduction.^6^ He argued that this system could take over, in the setting of ventricular disease, the function of the system described by Tawara. The potential significance of his findings was recognized by Hecht *et al*.^7^ when, in 1967, they summarized the ‘state of the art’ regarding the atrioventricular conduction axis. They recognized the significance of the point of penetration for demarcation of the atrial and ventricular components of the axis, but also argued in favour of a ‘penetrating’ bundle in addition to the ‘non-branching’ bundle.

## Describing the ventricular components of the conduction axis

As was described by Tawara,^1^ the node and the ventricular components can be distinguished using the point of insulation as the criterion.^1^ This then permits the ventricular parts of the axis to be separated into non-branching and branching components, with the branching part giving rise to the right and left bundle branches. It used to be considered that the branching bundle ‘bifurcated’. Massing and James^8^ showed that this was not the case, since in certain individuals, the axis itself continues as a ‘dead-end tract’.^9^ This feature is also important, since recently, the superior fascicle of the left bundle has been mistaken for the dead-end tract, described as the ‘retro-aortic root branch’.^10^ Equally important is to recognize that the fascicles of the left bundle, when the heart is viewed attitudinally, are located superiorly, septally, and inferiorly (*Figure 1A*). The trifascicular arrangement of the left bundle as shown by Tawara was subsequently endorsed by Kulbertus and colleagues,^11^ and then by Massing and James.^8^ Despite all this evidence, there are still some who continue to maintain that the left bundle is bifascicular.^12^ This may well be the case in species such as dogs and bovines,^13^ but there can be no doubt that, in the human heart, the left bundle branch is trifascicular.

## Putting the conduction axis back into the heart

Knowledge of the precise relationships of the conduction axis to the cardiac landmarks is obviously of importance to those seeking to pace the atrioventricular bundle, and also for those seeking to perforate the ventricular septum so as to achieve capture of its left fascicles.^14^ It is unfortunate, therefore, that those seeking to provide guidelines for these procedures should produce drawings with multiple anatomical inaccuracies. The drawing provided by Vijayaraman *et al*.^3^ shows a bifascicular left bundle branch descending the ventricular septum from the midpoint of the left atrioventricular junction. The drawing used by Jastrzebski *et al*.^14^ is based on a coronal section of the heart generated by Lynch and Jaffe, itself showing several anatomical inaccuracies. The recent guidelines of the European Heart Rhythm Associations,^15^ furthermore, have made used of our own previous accounts when discussing the relationship of the left bundle branch within the left ventricle, seemingly being unaware that we have now updated our own understandings,^5^ realizing that our initial dissection did not adequately show the intimate relationship of the superior fascicle of the left bundle to the nadir of the right coronary leaflet. It would also seem, however, that those seeking to pace the left bundle are unaware that, in the setting of ischaemic disease, it is lesions within the fascicles themselves that produce the abnormalities in conduction.^16^ Is it wise to seek to pace what could well be a diseased entity in the setting of conduction block? It is in this context that the findings of Mahaim are, perhaps, now of significance. It is entirely possible that pacing the non-branching or branching components of the ventricular conduction axis can activate the ‘paraspecific’ system described by Mahaim (*Figure* *1C* and *D*). Our own recent study has shown that, as suggested by Mahaim, these pathways are to be found in the majority of all normal hearts.^17^

## Guiding activities using the most reliable evidence

The accuracy achieved by Tawara^1^ in showing the location of the atrioventricular conduction axis is little short of astounding. Our own recent studies have endorsed his accounts.^2,5^ We submit that those seeking to guide others in the pursuit of pacing the ventricular conduction axis should now be in the position to make anatomically accurate drawings. They should also consider the possibility that pacing activates the superior septal pathways, as depicted by Mahaim,^6^ and endorsed by Hecht *et al*.^7^

## Data Availability

All relevant data are within the manuscript.
